# Hatching phase influences thermal preference of broilers throughout rearing

**DOI:** 10.1371/journal.pone.0235600

**Published:** 2020-07-06

**Authors:** João Batista Matos Júnior, Tamiris Iara Vicentini, Ayla Rosa Almeida, Viviane de Souza Morita, Sarah Sgavioli, Isabel Cristina Boleli

**Affiliations:** 1 Veterinary Medicine Sector, Faculty Marechal Rondon, Vilhena, Rondônia, Brazil; 2 Department of Animal Morphology and Physiology, School of Agricultural and Veterinary Sciences, São Paulo State University–UNESP, Jaboticabal, São Paulo, Brazil; 3 Brazil University, Descalvado, São Paulo, Brazil; Tokat Gaziosmanpasa University, TURKEY

## Abstract

Here we aimed for the first time to analyse whether opposite hatching patterns associated or not to high incubation temperature from day 13 to hatching interferes with the thermal preference and response of broilers to heat stress throughout the rearing period. Fertile eggs from 56-week-old broiler breeders (Cobb-500^®^) were used in a completely randomized trial with a 2x2 factorial arrangement (Short-Long and Long-Short hatching patterns: short time interval between internal and external pipping followed by long time interval between external pipping and hatching, and long time interval between internal and external pipping followed by short time interval between external pipping and hatching, respectively; and control and high incubation temperatures: 37.5°C and 39°C from the 13^rd^ day, respectively). Thermal manipulation from day 13 was chosen because it is known endocrine axes are already established at this time. At hatching, male chicks were reared in climatical chamber with 16 boxes, maintained at the temperature recommended for this strain, with 4 replicates of 18 chicks per treatment. Broilers with Long-Short hatching pattern and from eggs incubation at 37.5°C preferred the lowest ambient temperature at all analyzed ages, whereas broilers with Short-Long hatching pattern and from eggs incubated at 39°C preferred the highest temperatures from 21 days of age. Heat-exposed broilers showed increased respiratory frequency in all ages analyzed, which should have to contributed to maintainance of their rectal (body) temperature. The hatching patterns did not influence the feed intake, but broilers with Short-Long hatching pattern had better feed conversion, weight gain, and body weight. High incubation temperature reduced the feed consumption, as well as the weight gain and body weight by worsening the feed conversion. The results of this study reveal that hatching patterns associated or not to high incubation temperature influence the broiler thermal preference and heat response throughout the rearing period. Chicks with Long-Short and Short-Long hatching patterns should be reared separately, although this is not practical within a hatcher.

## Introduction

Hatching is a critical phase of the *in ovo* development of precocial birds. Briefly, hatching phase begins with coordinated head movements that allow the rupture of the inner shell membrane in the region of the egg air chamber (internal pipping), allowing the projection of the beak [1, 2] and at this time true lung respiration into the egg air chamber. Subsequently, a perforation is made in the shell, making it possible to project the beak out of the egg (external pipping). At last, counter-clockwise cracks are made around most of the shell circumference as chick rotates its entire body within the egg. Then, the shell cap is pushed off and the chick has to emerge [1]. Internal and external pipping times and the breaking of the eggshell have been associated to high O_2_ deficit and CO_2_ saturation [3, 4, 5] and high energy availability [6, 7, 8], respectively. The time interval between internal and external pipping shortens with the increase in the speed of PO_2_ reduction and PCO_2_ rise inside the egg air chamber [4]. At the same time, the chick needs to spend more energy to accomplish the external pipping and breaking of the eggshell [9, 10, 11, 12, 13], both of which involve an increase in gas exchange.

The embryonic development and growth of birds *in ovo* are temperature-dependent, since they can be blocked, activated, and have their speed altered by the ambient temperature. [14, 15, 16]. At the same time, gas exchange through the eggshell is directly related to the ambient temperature (gas diffusion law). Therefore, chick demand by energy and O_2_ [17, 8, 18]_,_ as well as exchange of O_2_ and CO_2_ throught the eggshell during hatching phase, respectively, must also be influenced by incubation temperature. Consequently, incubation time until internal pipping, external pipping, and actual hatching, and the time intervals between internal and external pipping, internal pipping and hatching, and external pipping and hatching might be influenced by incubation temperature manipulation, as already observed by some authors [17, 18, 19, 20].

It is known that incubation temperature might have consequences on the ambient temperature poultry require during the rearing phase. Heat exposure during egg incubation alters the thermal preference of turkeys, and laying and broiler chicks. Turkeys derived from eggs incubated at 38.5°C from the 7^th^ day onwards prefer high ambient temperatures [21]. Lohmann chicks subjected to intermittent heat stress (4 hours/day at 40°C) in the period between the 14^th^ to 18^th^ day of incubation prefer low ambient temperatures up to the 7^th^ day of life [22]. In previous studies, we found that broiler chicks from eggs incubated at high temperature (39°C) from day 13 of incubation (fetal phase) have thinner and more vasculated skin, that may help chicks to have greater heat loss and to maintain body temperature, and prefer higher rearing temperature until the 21^st^ day of life compared to broilers from egg incubation at usual (37.5°C) and cold (36°C) temperature [23, 24].

The time intervals between the internal and external pipping and the external pipping and actual hatching form a transitional phase between purely allantoic and exclusively pulmonary respiration, essential for chick survival outside the egg. In previous study, we verified that chicks with short time interval between internal and external pipping had long time interval between external pipping and hatching and vice-versa, these short-long and long-short patterns of hatching phase affected the chick quality and blood response. According to results, 84% of the chicks presenting Long-Short hatching pattern had no physical problems with feathering, eyes, umbilical region, legs, alantoic cord, and yolk-sac incorporation, whereas only 50% of the chicks with Short-Long hatching pattern did not show physical problems [25]. Also, chicks with Short-Long hatching pattern showed higher red blood counts (RBC), hemoglobin values (Hg) and mean corpuscular hemoglobin concentration (MCHC), indicating higher gas exchange potential compared to chicks with Long-Short hatching pattern [25]. Thus, it is likely that prenatal factors affecting hatching length, for example, incubation temperature, may also influence the ability of broilers with distinct hatching pattern cope with stress. To our knowledge, no research is available in which attempts have been made to assess potential effects of hatching pattern and of the association between hatching pattern and incubation temperature on the thermal preference and thermal tolerance of broilers during rearing.

We hypothesize that hatching pattern affects thermal preference and response to thermal challenge of broilers throughout the rearing phase, and that its effects can be altered by incubation temperature manipulation. To test this hypothesis, this study analyzed: (i) whether and for how long distinct hatching patterns affect thermal preference and response to thermal challenge of broilers throughout the rearing phase; and (ii) whether and how high incubation temperature manipulation during fetal development affects the hatching pattern effects on the broiler thermal preference and response to thermal challenge. The answers to these questions may contribute to further the current understanding on the importance and effects of hatching pattern associated or not to incubation temperature manipulation on the post-hatching ontogenetic development and their potential use to improve poultry production.

## Materials and methods

The experimental protocol of this study was approved by the local Ethics Committee on Animal Use (CEUA, Protocol N^o^. 022383/12), of the College of Agricultural and Veterinary Sciences, of the São Paulo State University (UNESP), Jaboticabal, São Paulo, Brazil.

### Experimental conditions

In this study, the experimental arrangement followed a factorial 2x2, with 2 incubation temperatures from the 13^th^ day (37.5°C and 39°C) and two hatching patterns [Short-Long and Long-Short hatching patterns: short time interval between internal and external pipping (2-8h10min) followed by long time interval between external pipping and hatching (20-26h), and long time interval between internal and external pipping (11-18h) followed by short time interval between external pipping and hatching (6-10h), respectively]. For this, about 900 fertile eggs from 56-week-old broiler breeder flocks (Cobb500^®^) were obtained from a commercial hatchery (Globoaves, Itirapina, São Paulo, Brazil). They were individually weighted and homogeneously distributed by weight (65-70g) over 10 incubators (Premium Ecológica IP120, Belo Horizonte, MG, Brazil) (N: 90 eggs/incubator) with automatic control of temperature and egg turning. All incubators were kept at 37.5°C (the usual incubation temperature for broilers) from the 1^st^ to the 12^th^ day of incubation. From day 13 until hatching, thermal programming was applied and 5 incubators had their temperature raised to 39°C, whereas the other 5 incubators had their temperature maintained at 37.5°C (control). Thermal programming from the 13^rd^ day of incubation was based on establishment of the hypothalamus-pituitary-thyroid axis already at this age [26, 27] It was maintained until hatching. In all incubators, relative humidity was maintained at 60% throughout the incubation period (504 hours), including hatching phase, to eliminate effects on embryonic development and mortality. Egg turning (45° of rotation, each hour) was maintained until the 18^th^ day of incubation. Eggshell temperature (EST) was measured every 30 min from onset of incubation until day 19 using thermosensors (Alutal, PT100, 2x1.5mm, accuracy: 0.15°C; Sao Paulo, Brazil) attached to the shells at the equatorial region of the eggs, covered with Styrofoam (2x2mm), and attached with regular cellophane tape (3M)(2x3mm). EST was stored in data loggers connected to a computer. The average EST from day 13 was 37.5±1.93°C and 37.44±2.01°C for eggs incubated at 37.5°C that originated male chicks with Long-Short (N = 12 eggs) and Short-Long (N = 7 eggs) hatching patterns, respectively; and 39±1.83°C and 38.9±1.59°C for eggs incubated at 39°C that originated male chicks with Long-Short (N = 8 eggs) and Short-Long (N = 9 eggs) hatching patterns, respectively. The Long-Short and Short-Long hatching patterns were established based in previous study, showing chicks with short time interval between internal and external pipping had long time interval between external pipping and hatching and vice-versa [25], and in additional observations. At 19 days of incubation (456h), all eggs were checked by candling and moved to hatching baskets, which was placed in the same incubators. Infertile eggs and eggs containing dead embryos were removed. To determine the time intervals between external pipping and actual hatching (Short and Long), all eggs were continuously monitored from day 19 by direct visual observation through the acrylic cover of the incubators, writing down the day and time of external pipping (eggs with cracked shell and beak projected out of the shell) and hatching (chick emerged from the eggshell, still wet and panting) for each of the eggs. Incubation period was considered finished with 504h. In eggs incubated at 37.5°C, the chicks hatched from 486 to 502h of incubation, the hatching window lasted 16h, the overall hatching time was 496h, and the hatching rate was 85%. Under egg incubation at 39°C, the hatching rate was 83%, the chicks hatched from 477 to 492h, the overall hatching time was 487h, and the hatching window was 15h.

### Rearing

At 495h of incubation, all the chicks were removed from the hatchers and sexed by examining the feathers [28] Then, they were maintained in chick brooders with electric heating done by infrared lamps (40W) (Premium Ecológica 1200, BH, MG, Brazil), receiving water *ad libitum* to avoid dehydration, whereas the chicks presenting Short-Long and Long-Short hatching patterns were separeted (approximately 60min). Only chicks born from 477 to 492h of incubation were chosen for rearing, as they presented the hatching patterns established in the experimental design of this study in a short hatching window to avoid fasting effects. Then, 72 male chicks (hatched from 477 to 492h of incubation) from each treatment were housed in climatic chamber with automatic control of temperature and light period (light:dark = 22h:2h) [23], containing 16 boxes (1.5x 2.50 m, 18 chicks per box), kept at recommended temperature for the strain [28]. The temperature and relative humidity in the chamber were registered three times a day during the experimental period, and for this we used two digital thermo-hygrometers located at equidistant points of the chamber. The average weekly values of temperature and relative humidity in the chamber were 33.4°C and 67%, 29.5°C and 76%, 27.2°C and 68%, 25.3°C and 64%, 23°C and 65%, and 21°C and 60%, from the first to the sixth week of the experiment, respectively. Throughout the experimental period, water and feed were provided *ad libitum*. The ratios were based on corn and soybean meal, and formulated in accordance with the requirements established for broilers for initial (1 to 21 days: ME 2,883Kcal/kg, CP 21.27%) and growth (22 to 42 days: ME 3,121Kcal/kg, CP 18.86%) phases [29]. The chicks were vaccinated against Marek and poxvirus disease still in the hatchery, against infectious bursal disease and Newcastle on the 8^th^ day of age and against Gumboro disease on the 18^th^ day.

### Thermal preference tests

The thermal preference test was based on the methodology used previously by other authors [22, 30] and adapted for ours studies [23, 24]. Briefly, two thermal preference test chambers with similar dimensions (length x width x height: 50 x 160 x 60 cm), thermal gradient system, and registration of temperature and chicken location were used. Both chamber presented a temperature gradient (from 19 to 40°C along the length of the chambers) generated by two thermal resistances and one cold air intake located at opposite ends of the chambers, and recorded by 12 temperature sensors distributed in equidistant manner along of the length of one of the sides of the chamber. The position and displacement of the poultry inside the chambers were recorded by 12 infrared sensors distributed along of the length of the chambers on the opposite to the thermal sensors. Data for temperature and bird position were registered and stored per minute, by means of a software program specifically developed for acquisition of thes225)e data, which is coupled to a computer. According to a previous study [23], thermal preference tests were run simultaneously in both chambers at the 1^st^, 7^th^, 14^th^, 21^st^, 28^th^, and 35^th^ post-hatching day from 7:30 to 16: 30h, using two birds at the same time within each test chamber (n = 12 birds/treatment). Two chicks from the same treatments and age were put together into each test chamber, because preliminary study had shown that using one chick per test resulted in immobility of the chicks and absence of exploration of the thermal gradient within the chambers. However, when two chicks were used at the same time, such immobility did not occur, and both chicks explored the temperature gradient [23]. Thermal tests were not realized at the 42^nd^ day of age, because 42-day-old birds remained immobile and did not explore the temperature gradient of the test chambers. Thermal preference tests lasted 90 minutes divided into two phases: initial 30 min for ambient recognition by the birds and 60 minutes for positioning and holding at a temperature of preference. After completion of the tests, from the software records was determined the ambient temperature preferred by each bird, which corresponded to ambient temperature in which the bird remined for the longest time. If the birds remained for a long time at two or more temperatures, the average ambient temperature was calculated. Subsequently, the birds were identified and again housed in the original climate chambers to be used in the thermal challenge tests on the following day and also to maintain the bird number per replicate. The rectal temperature of the birds was measured with a digital thermometer prior and after the thermal preference tests.

### Thermal challenge tests

Thermal challenge was used in this study to verify whether broilers with Long-Short and Short-Long HP respond diferently to heat exposure during rearing and whether incubation at 39°C during fetal phase made these birds more tolerant to heat. The birds were subjected to thermal challenge tests according to methodology previously established [23, 24], as follows: we used two acrylic climate chambers (80 x 80 x 80 cm) containing automatic control of temperature, relative humidity and air movement, making it possible to vary the ambient temperature from 15 to 40°C. Thermal challenge tests were performed on the 2^nd^, 8^th^, 15^th^, 22^nd^, 28^th^, and 36^th^ post-hatching day, using the birds submitted to thermal preference tests conducted the day before. We used three birds per treatment each test, totaling 12 birds/treatment/age [23]. The thermal challenge test consisted of three consecutive phases of 45 minutes duration each, totalizing 135 minutes. During the first phase of the thermal challenge test, the birds were exposed to their preferred temperature established in the preference test in the previous day. During the thermal challenge phase, the birds were exposed to temperature 5°C higher than their preferred temperature. After the thermal challenge, again the birds were exposed to preferred temperature. The average time it took the thermostat to increase or reduce the temperature of the challenge chambers by 5°C degrees was approximately 4 minutes, which was included in the total 45 minutes duration of the last two phases to the challenge tests, respectively. At the end of each phase, respiratory frequency (RF_;_ resp.mov.min^-1^) of the birds was determined by direct observation. The observer was always the same. After this registration, the rectal temperature of the birds was measured using a digital thermometer for veterinary use (Incoterm®, model 5198, range between 34 and 44 and 0,1°C error limit). Different birds were used on each test day to prevent the bird`s adaptation to the chambers and to avoid thermal conditioning at high temperatures during the functional maturation period of the thermoregulaty system of the birds, which occurred approximately until day 10 of age [31, 32].

### Broiler performance

Feed intake, weight gain, feed conversion and mortality were determined for the total rearing period (from day 1 to 42 of age). Amounts of feed offered to each replicate were recorded weekly, and uneaten feed in each replicate was weighed. Weight gain was determined by the difference between body weight at the beginning and end of the rearing period. Feed conversion was calculated by dividing the amount of feed intake by the body weight gain (grams per gram). Broiler mortality was verified. Broiler weight was measured on day 42 of age. All data were determined on a replicate basis.

### Statistical analyses

The effects of the hatching pattern (HP_:_ Long-Short and Short-Long), incubation temperature (IT: 37.5°C and 39°C) and the interaction between both (HP x IT) on the preferred temperature, the rectal temperature before and after the thermal preference tests, respiratory frequency, and rectal temperature during the thermal challenge tests (before, at the end the thermal challenge, and after the thermal challenge), obtained at different ages, were analyzed using the experimental model: Y_ijk_ = *μ* + (HP)_i_ + IT_J_ + (HP x IT)_ij_ + e_ijk_, in which Y = dependent variable, *μ* = overall mean, and e = residual error. Feed intake, weight gain, feed conversion, and body weight wer analyzed with the same model. In addition, the rectal temperatures and the respiratory frequency obtained in the three phases of the thermal challenge tests (before, at the end and after the thermal challenge) were compared within each treatment and age, using the model: Y = μ + thermal challenge phase + e. All data were submitted to analysis of variance by the one-way ANOVA using the GLM procedure of SAS [33], and statistical significance was set at 5%. In case of significant interactions, the Tukey test was applied for comparison among means.

## Results

This study examined whether the hatching pattern (HP) and the incubation temperature (IT) in the fetal phase affect the thermal preference and the response to heat stress of broilers throughout rearing.

### Effects of HP and IT on thermal preference

The results of the tests to determine the broiler`s thermal preference throughout rearing are shown in Table 1. We found significant interactions between HP and IT on the thermal preference at the 1^st^, 7^th^, 14^th^, and 35^th^ day of age (*P* ≤ 0.05). On the first day, chicks derived from eggs incubated at 37.5°C and presenting Long-Short HP preferred the lowest temperatures. Chicks from all the other treatments showed similar rearing temperature preferences (*P* > 0.05). At the 7^th^ day, chicks derived from eggs incubated at 37.5°C and with Long-Short HP continued to prefer the lowest whereas those presenting Short-Long HP favored the highest temperatures compared with the chicks from the other treatments. Similar rearing temperatures were elected by the chicks derived from eggs incubated at 39°C of Short-Long and Long-Short HP (*P* > 0.05). At the 14^th^ days of age, chicks derived from the 37.5°C egg incubation and with Long-Short HP persisted with their preference for low temperatures. Interestingly, at this stage, chicks from 39°C incubation and presenting Short-Long HP also preferred low whereas those presenting Long-Short HP favored the highest rearing temperatures. At the 21^st^ and 28^th^ days, no significant interactions between HP and IT were found for the preferred temperature (*P* > 0.05), but significant effects of HP and IT were observed (*P* ≤ 0.05). At both ages, broilers showing Short-Long HP and broilers from eggs incubated at 39°C preferred higher rearing temperatures than broilers with Long-Short HP and from eggs incubated at 37.5°C, respectively. At day 35, significant interactions between HP and IT were detected (P≤0.05). Once again, broilers of Long-Short HP and egg incubation at 37.5°C preferred the lowest temperatures and broilers of Short-Long HP derived from eggs incubated at 39°C preferred the highest temperatures (*P >* 0.05). Taken together, independently of the existence of significant interactions between HP and IT, broilers of Long-Short HP derived from eggs incubated at 37.5°C always preferred the lowest temperatures and broilers of Short-Long HP derived from eggs incubated at 39°C favored the highest rearing temperatures from day 21 onwards.

**Table 1:** Thermal preference (°C) of broilers with Short-Long and Long-Short hatching pattern obtained from eggs submitted to usual (37.5°C) or high (39°C) incubation temperature from day 13 of incubation till hatching.

Treatments	Days of Age					
	1^st^	7^th^	14^th^	21^st^	28^th^	35^th^
HP _X_ IT						
Long-Short-37.5°C	32.50±0.53^b^	28.37±1.50^c^	27.12±0.83^c^	25.87±0.35	24.87±0.35	23.25±0.46^c^
Long-Short-39°C	33.87±0.35^a^	30.25±0.46^b^	29.37±0.91^a^	26.62±0.74	25.50±0.53	24.62±0.51^b^
Short-Long-37.5°C	33.75±0.70^a^	31.12±0.35^a^	28.25±0.46^b^	26.12±0.64	25.12±0.35	24.87±0.35^b^
Short-Long -39°C	34.25±0.46^a^	30.00±0.53^b^	27.75±0.46^b^^c^	27.62±0.74	26.00±0.00	25.50±0.53^a^
Hatching Pattern (HP)^1^						
Long-Short	33.18±0.83	29.31±1.44	28.25±1.43	26.25±0.68^b^	25.18±0.54^b^	23.93±0.85
Short-Long	34.00±0.63	30.56±0.72	28.00±0.51	26.87±1.02^a^	25.56±0.51^a^	25.18±0.54
Incubation temperature (IT)						
37.5°C	33.12±0.88	29.75±1.77	27.68±0.87	26.00±0.51^b^	25.00±0.36^b^	24.06±0.93
39°C	34.06±0.44	30.12±0.50	28.56±1.09	27.12±0.88^a^	25.75±0.45^a^	25.06±0.68
*P*-value						
HP	0.0002	0,0003	0.3216	0.0101	0.0072	<0.0001
IT	<0.0001	0.2226	0.0015	<0.0001	<0.0001	<0.0001
HP x IT	0.0270	<0.0001	<0.0001	0.1091	0.3423	0.0329
CV (%)	1.57	2.84	2.49	2.41	1.44	1.92

CV: coeficent of variation.

^a-c^: means followed by the same superscript letter in the columns do not differ from each other (*P* ≤ 0.05).

^1^Time intervals between pippings and between external pipping and actual hatching (Short-Long: 2-8h10min+20-26h, Long-Short: 11–18+6-10h). Mean±SD, n = 12 birds/ treatment.

### Effects of HP and IT on rectal temperature before and after the thermal preference tests

Table 2 contains data of rectal temperatures presented by the broilers before and after the thermal preference test. There was no significant interaction between HP and IT for any of the analyzed ages for rectal temperatures before the thermal preference test. However, a significant effect of IT was observed on rectal temperature of the chicks at the 1^st^ and 14^th^ day of age, where chicks presented higher rectal temperatures when egg incubation occurred at 37.5°C (*P* ≤ 0.05). None of the analyzed ages presented significant interaction between HP and IT for rectal temperatures after the thermal preference test. There was a significant effect of IT on the 14^th^ and 35^th^ day of age and HP on day 35 (*P* ≤ 0.05). In these ages, the rectal temperature was higher in broilers from eggs incubated at 37.5°C than at 39°C, and in broilers with Short-Long as opposed to Long-Short HP.

**Table 2:** Rectal temperature before and after the thermal preference test of broilers with Short-Long and Long-Short hatching pattern obtained from eggs submitted to usual (37.5°C) or high (39°C) incubation temperature from day 13 of incubation till hatching.

Treatments	Days of Age					
	1^st^	7^th^	14^th^	21^st^	28^th^	35^th^
	Before the Thermal Preference Tests					
Hatching Pattern (HP)^1^						
Long-Short	38.8±0.9	40.8±0.4	41.7±0.3	42.0±0.5	42.1±0.2	42.1±0.3
Short-Long	38.7±0.7	40.8±0.2	41.7±0.4	41.9±0.5	42.0±0.2	42.1±0.2
Incubation Temperature (IT)						
37.5°C	39.3±0.5^a^	40.9±0.3	41.9±0.4^a^	41.9±0.4	42.0±0.3	42.0±0.2
39°C	38.1±0.5^b^	40.8±0.3	41.5±0.2^b^	41.9±0.5	42.1±0.1	42.1±0.3
*P*-value						
HP	0.3798	0.6476	0.4650	0.6618	0.3612	0.5829
IT	<0.0001	0.4939	0.0047	0.8839	0.2160	0.1880
HP x IT	0.8045	0.9089	0.9165	0.9418	0.4539	0.9373
CV (%)	1.27	0.75	0.80	1.14	0.49	0.52
After the Thermal Preference Tests						
Hatching Pattern (HP)^1^						
Long-Short	40.1±0.5	40.7±0.4	41.2±0.4	41.7±0.4	41.5±0,3	41.5±0.2^b^
Short-Long	40.1±0.7	40.9±0.4	41.4±0.3	41.7±0.3	41.7±0,6	41.9±0.3^a^
Incubation Temperature (IT)						
37.5°C	40.3±0.5	40.8±0.4	41.5±0.3^a^	41.6±0.3	41.6±0.6	41.8±0.4^a^
39°C	39.9±0.7	40.8±0.4	41.2±0.3^b^	41.8±0.4	41.5±0.4	41.6±0.3^b^
*P*-value						
HP	0.9507	0.2248	0.0602	0.9584	0.4222	0.0002
IT	0.1159	0.7922	0.0056	0.1144	0.8833	0.0419
HP x IT	0.1300	0.6610	0.0782	0.0938	0.5583	0.7491
CV (%)	1.41	0.97	0.65	0.80	1.14	0.65

CV: coefficent of variation.

^1^Time intervals between pippings and between external pipping and actual hatching (Short-Long: 2-8h10min+20-26h, Long-Short: 11–18+6-10h).

^a-b^: means followed by the same superscript letter in the columns do not differ from each other (*P* ≤ 0.05). Mean±SD, n = 12 birds/ treatment.

### Effects of HP and IT on respiratory frequency (RF) in the three phases of the thermal challenge test

**Fig 1:**
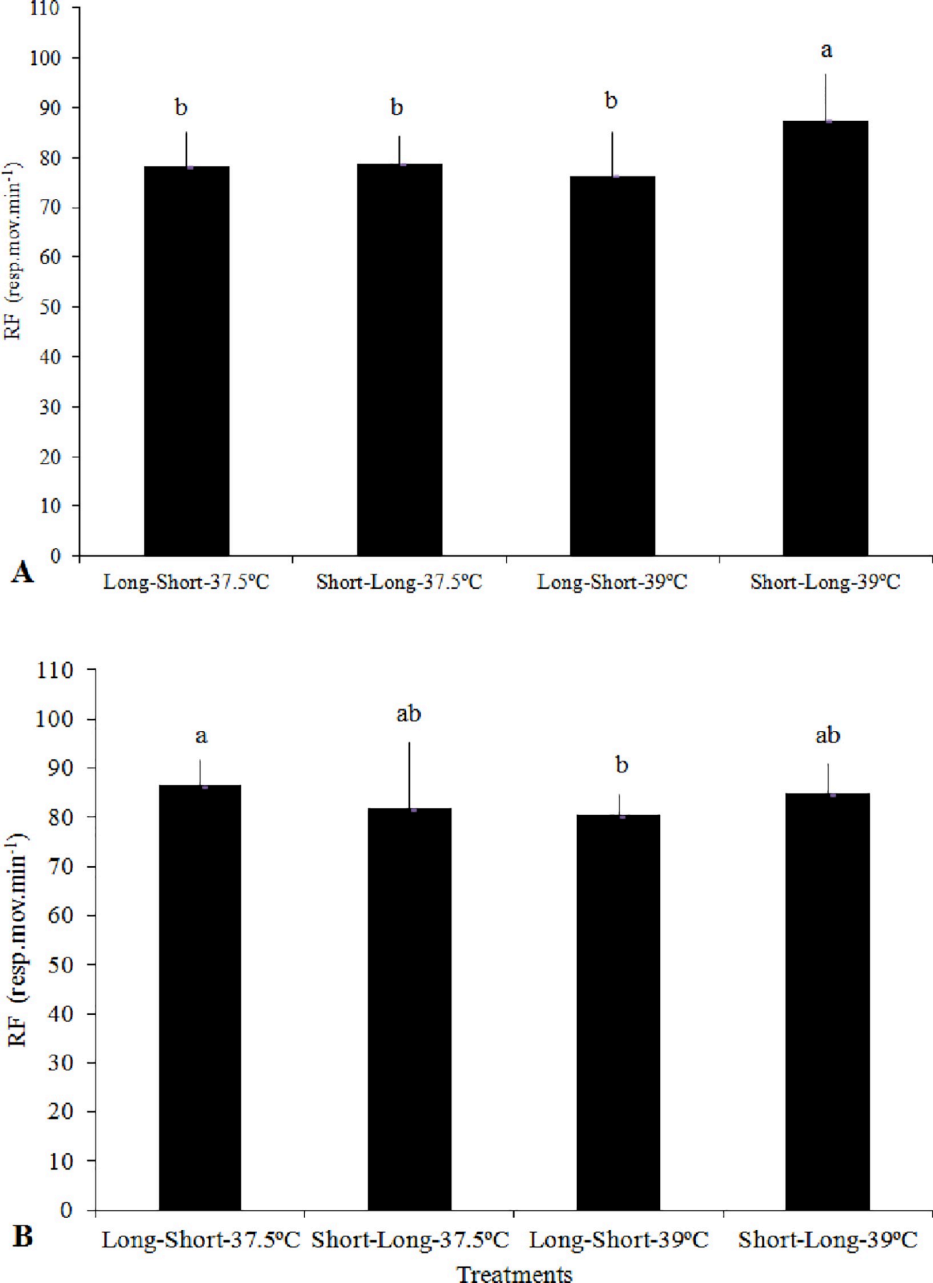
Respiratory frequency (RF) of broilers aged 8 (A) and 29 (B) days, at the end of the thermal challenge phase, with the hatching pattern (Short-Long or Long-Short) and the incubation temperature (37.5°C or 39.0°C from day 13). Short-Long and Long-Short: Time intervals between pippings and between external pipping and hatching (2-8h10min+20-26h and 11–18+6-10h, respectively). a-b: means with similar letters do not differ from each other (Tukey test, *P* ≤ 0.05).

The first phase of the thermal challenge test consisted of exposing the broilers to their preferred rearing temperatures for 45 minutes and then taking the relevant measurements. According to Table 3, the RF presented by broilers at the first phase of the thermal challenge test were not affected by the HP and IT. We did not find significant interactions between these factors at any of the analyzed ages. The RF of the broilers at the end of the thermal challenge were affected by HP at the 8^th^ and 15^th^ days, by interactions between the HP and IT at the 8^th^ and 29^th^ days of age, and by IT at the 36^th^ day. According to interactions between HP and IT, as shown in Fig 1A, at the 8^th^ day, broilers with Short-Long HP derived from eggs incubated at 39°C presented higher RF than the broilers from the other treatments. At day 15, the RF was lower in broilers with Short-Long HP than in broilers with Long-Short HP. At day 29, the RF differed between the broilers with Long-Short HP from eggs incubated at 37.5°C and 39°C, being higher in the former group than in the latter (Fig 1B). At the 36^th^ day, the RF was higher in broilers from eggs incubated at 39°C than in those broilers from egg incubation at 37.5°C. At the final phase of the thermal challenge test, when broilers were returned to the preferred rearing temperatures, we found that HP and IT did not significantly affect their RF (Table 3).

**Table 3:** Respiratory frequency (RF, resp.mov.min^-1^) of broilers with Short-Long and Long-Short hatching pattern obtained from eggs submitted to usual (37.5°C) or high (39°C) incubation temperature from day 13 of incubation till hatching.

Treatments	Days of Age																					
	2^nd^		8^th^				15^th^				22^nd^				29^th^				36^th^			
	RF at the preferred rearing temperature before thermal challenge																					
Hatching Pattern (HP)^1^																						
Long-Short	57.6±6.5	72.2±6.4				67.3±7.3				64.9±5.6				60.4±4.7				53.3±4.3				
Short-Long	56.0±3.9	72.5±4.9				62.9±6.4				66.2±5.6				61.8±8.2				52.4±4.5				
Incubation Temperature (IT)																						
37.5°C	56.7±5.5			70.9±5.3				66.0±8.7				65.6±5.5				61.6±6.2					52.4±4.1	
39°C	56.9±5.2			73.9±5.7				64.2±5.2				65.6±4.8				60.7±7.2					53.3±4.8	
*P*-value																						
HP	0.3862			0.8627				0.0661				0.4366				0.5632					0.5595	
IT	0.8905			0.1267				0.4522				1.0000				0.6995					0.5595	
HP x IT	0.1895			0.9540				0.8503				0.1249				1.0000					0.5595	
CV (%)	9.36			7.80				10.76				7.74				11.20					8.54	
	RF at the end of the thermal challenge^2^																					
Hatching Pattern (HP)^1^																						
Long-Short	68.9±10.8				77.3±7.8^b^				72.7±4.9^a^				79.1±6.7				83.6±5.5					84.0±6.0
Short-Long	65.3±11.3				83.1±8.7^a^				69.3±4.1^b^				76.9±8.5				83.3±6.3					82.9±4.7
Incubation Temperature (IT)																						
37.5°C	68.7±11.6				78.4±6.2				70.7±5.8				77.1±7.2				84.2±6.2					81.1±4.3^b^
39°C	65.6±10.6				82.0±10.5				71.3±3.9				78.9±8.1				82.7±5.5					85.8±5.4^a^
*P*-value																						
HP	0.3506			0.0337				0.0333				0.3971				0.9044				0.5011		
IT	0.4133			0.1815				0.6099				0.4973				0.4031				0.0074		
HP x IT	0.9065			0.0488				0.3970				0.4973				0.0161				0.3479		
CV (%)	16.77			9.73				6.49				9.95				6.60				5.87		
	RF at the preferred rearing temperature after thermal challenge																					
Hatching Pattern (HP)^1^																						
Long-Short	58.1±6.3			68.2±7.9				65.1±4.9				64.0±3.6				59.8±5.2				54.0±5.0		
Short-Long	55.3±4.2			69.6±5.2				63.8±3.8				64.2±4.0				60.0±5.7				54.2±4.4		
Incubation Temperature (IT)																						
37.5°C	57.3±5.8			69.3±5.1				64.0±4.8				63.6±3.6				59.6±6.7				52.7±3.7		
39°C	56.0±5.1			68.4±8.0				64.9±4.0				64.7±3.9				60.2±3.8				55.6±5.1		
*P*-value																						
HP	0.1458			0.5457				0.3725				0.8634				0.9059				0.8824		
RT	0.4612			0.6866				0.5507				0.3923				0.7232				0.0615		
HP x IT	0.1458			0.0762				0.3725				0.3923				0.9059				0.1892		
CV (%)	9.31			9.50				6.86				5.99				9.34				8.26		

CV: coefficent of variation.

^1^Time intervals between pippings and between external pipping and actual hatching (Short-Long: 2-8h10min+20-26h, Long-Short: 11–18+6-10h). mean±SD, n = 12 birds/ treatment.

^2^Broilers exposed to ambient temperature 5°C higher than the preferred temperature for 45 min.

^a-b^: means followed by the same superscript letter in the columns do not differ from each other (*P* ≤ 0.05).

### Effects of HP and IT on the rectal temperature in the three phases of the thermal challenge test

**Fig 2:**
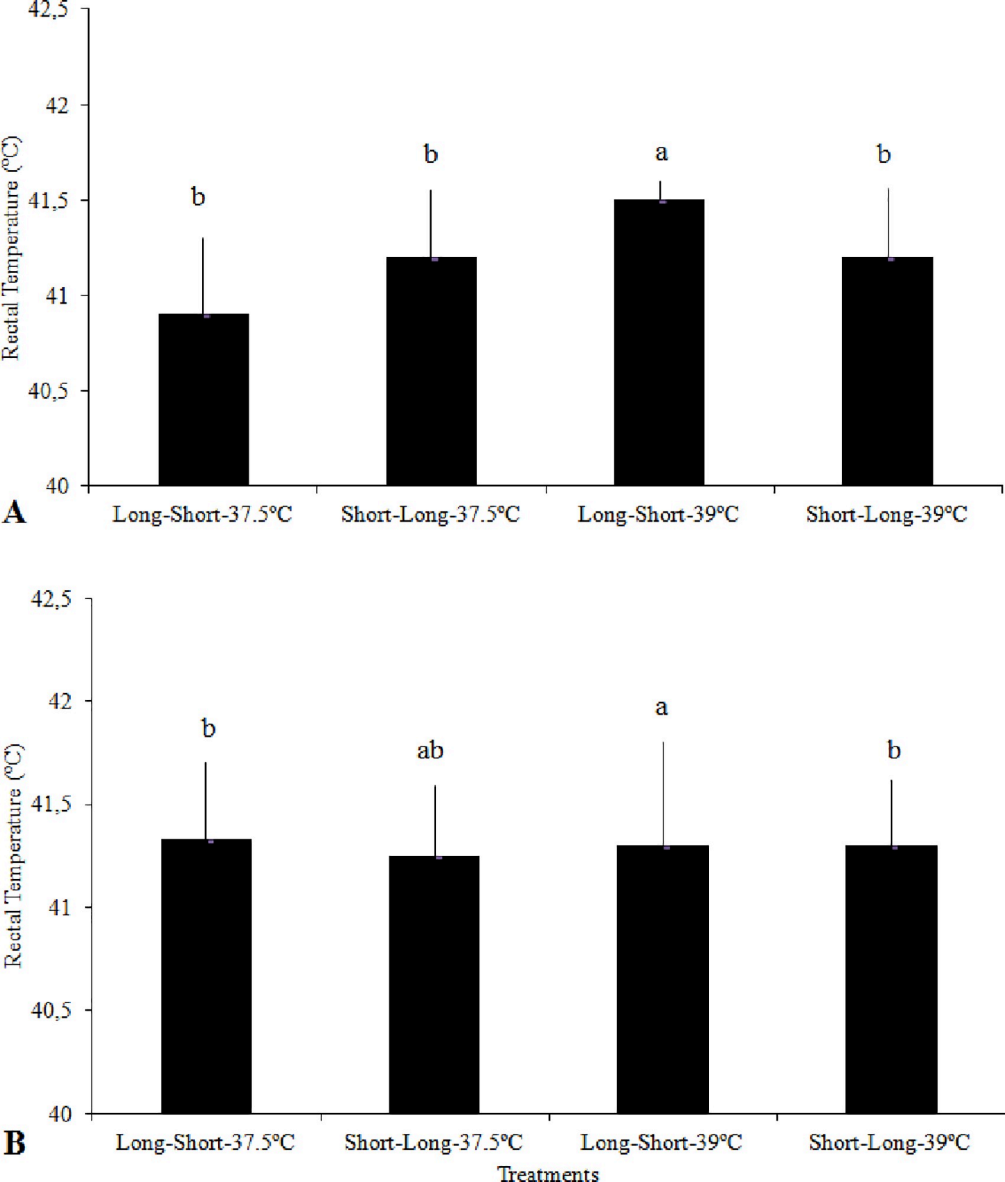
Rectal temperatures (RT) of broilers at the preferred temperature before thermal challenge (A) and at the end of the thermal challenge (B), with the interaction between hatching pattern (HP: Short-Long or Long-Short) and incubation temperature (37.5°C or 39°C from day 13). a-b: means with similar letters do not differ from each other (Tukey test, *P* ≤ 0.05). Short-Long and Long-Short: time intervals between pippings and between external pipping and hatching (2–8,2+20-26h and 11–18+6-10h, respectively).

In this phase, the rectal temperature of broilers was influenced by IT at the 2^nd^ day of age and an interaction between HP and IT was observed at the 36^th^ day of age (Table 4). At the 2^nd^ day, the rectal temperatures were higher in broilers from eggs incubated at 39°C than in those whose eggs were incubated 37.5°C. At day 36, broilers with Long-Short HP from eggs incubated at 39°C presented the highest rectal temperature whereas no significant difference were found among broilers from the other three treatments (Fig 2A).

**Table 4:** Rectal temperatures (RT) of broilers with Short-Long and Long-Short hatching pattern obtained from eggs submitted to usual (37.5°C) or high (39°C) incubation temperature from day 13 of incubation till hatching in the three phases of the thermal challenge test.

	Days of Age					
Treatments	2^nd^	8^th^	15^th^	22^nd^	29^th^	36^th^
	RT at the preferred rearing temperature before thermal challenge (°C)					
Hatching Pattern (HP)^1^						
Long-Short	40.2±0.3	40.9±0.3	41.1±0.5	41.3±0.2	41.2±0.3	41.2±0.4
Short-Long	40.1±0.4	40.9±0.1	41.0±0.4	41.2±0.3	41.0±0.3	41.2±0.3
IT						
37.5°C	40.0±0.4^b^	40.9±0.2	41.0±0.4	41.3±0.2	41.1±0.3	41.1±0.4^b^
39°C	40.3±0.3^a^	40.8±0.3	41.2±0.5	41.3±0.3	41.1±0.3	41.4±0.3^a^
*P*-value						
HP	0.6248	0.9860	0.7165	0.2910	0.0873	0.7125
IT	0.0240	0.5862	0.1736	0.8600	0.4856	0.0108
HP xIT	0.2083	0.7649	0.0566	0.8711	0.1095	0.0063
CV (%)	0.84	0.56	1.10	0.69	0.69	0.76
	RT at the end of the thermal challenge^2^ (°C)					
Hatching Pattern (HP)^1^						
Long-Short	40.4±0.2	40.8±0.3	41.3±0.5	41.4±0.2	41.2±0.4	41.3±0.4
Short-Long	40.4±0.3	40.9±0.2	41.1±0.4	41.5±0.3	41.1±0.4	41.3± 0.3
Incubation Temperature (IT)						
37.5°C	40.3±0.2^b^	40.9±0.3	41.1±0.3	41.5±0.3	41.0±0.4	41.3±0.3
39°C	40.5±0.2^a^	40.8±0.2	41.3±0.6	41.4±0.2	41.2±0.4	41.3±0.4
*P*-value						
HP	0.3277	0.4742	0.1622	0.6728	0.3688	0.6353
IT	0.0004	0.3308	0.1622	0.4453	0.2860	0.9655
HP x IT	0.3277	0.5577	0.0263	0.4453	0.6363	0.8969
CV (%)	0.50	0.62	0.98	0.68	0.93	0.92
	RT at the preferred reading temperature after thermal challenge (°C)					
Hatching Pattern (HP)^1^						
Long-Short	40.1±0.3	40.7±0.3	40.7±0.2	41.1±0.2	41.0±0.3	41.0±0.5
Short-Long	40.2±0.3	40.7±0.3	40.7±0.3	41.0±0.4	40.9±0.3	41.0±0.4
Incubation Temperature (IT)						
37.5°C	40.1±0.3	40.8±0.3	40.8±0.3	41.2±0.3^a^	40.9±0.3	41.0±0.3
39°C	40.1±0.3	40.7±0.4	40.7±0.3	40.9±0.3^b^	41.0±0.4	40.9±0.5
*P*-value						
HP	0.3719	0.8837	0.7312	0.1994	0.6360	1.0000
IT	0.8532	0.4665	0.4932	0.0174	0.7762	0.2476
HP x IT	0.3191	0.4098	0.6472	0.4502	0.7048	0.5344
CV (%)	0.75	0.83	0.70	0.73	0.85	1.03

CV: coefficent of variation.

^1^Time intervals between pippings and between external pipping and actual hatching (Short-Long: 2-8h10min+20-26h, Long-Short: 11–18+6-10h). mean±SD, n = 12 birds/ treatment.

^2^Broilers exposed to ambient temperature 5°C higher than the preferred temperature for 45 min.

^a-b^: means followed by the same superscript letter in the columns do not differ from each other (*P* ≤ 0.05).

The actual thermal challenge phase consisted in exposing the broilers to temperatures 5°C above their preferred rearing temperatures for 45 minutes and then taking the relevant measurements. At the 2^nd^ day of age, broilers subjected to thermal challenge were affected by IT. At this age, the rectal temperature remained higher in broilers from eggs incubated at 39°C than those from eggs incubated at 37.5°C. An interaction between HP and IT was seen at the 15^th^ day (Table 4). Rectal temperatures were higher in the broilers with Long-Short HP than in those with Short-Long HP of eggs incubated at 39°C, and than the broilers with Long-Short HPderived from eggs incubated at 37.5°C (Fig 2B).

The third and final phase of the thermal challenge test consisted in returning the broilers to their preferred rearing temperatures for 45 minutes and then taking the relevant measurements. The rectal temperatures after thermal challenge were affected by IT at the 22^nd^ day of age, and were higher in broilers whose eggs were incubated at 37.5°C than those derived from eggs incubated at 39°C (Table 4).

### Variation of the respiratory frequency (RF) in the three phases of the thermal challenge test

**Fig 3:**
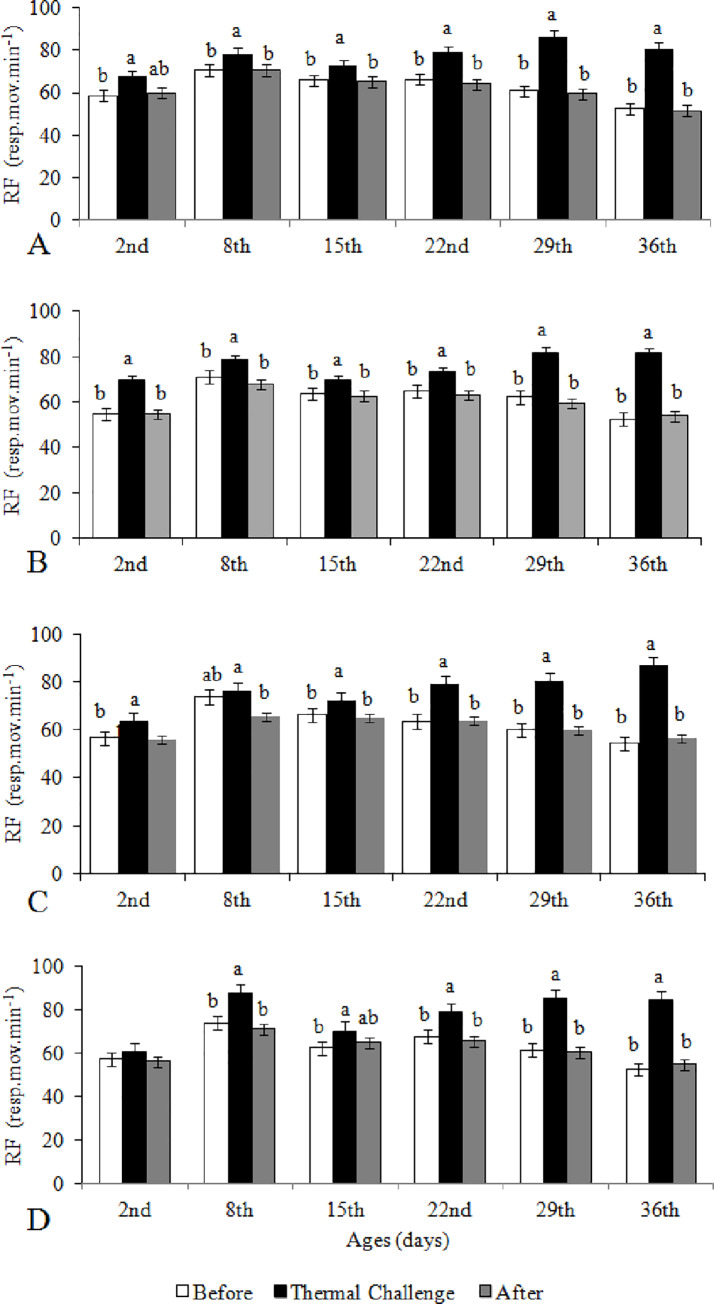
Respiratory frequency (RF) of broilers from eggs incubated at 37.5°C (A,B) and 39°C (C, D) with Long-Short (A, C) and Short-Long (B, D) hatching pattern obtained at the preferred temperature before thermal challenge, at thermal challenge (5°C above thermal preference for 45 min), and upon return to thermal preference after thermal challenge, according to age. Short-Long and Long-Short: time intervals between pippings and between external pipping and hatching (2–8,2+20-26h and 11–18+6-10h, respectively). a-b: means with similar letters do not differ from each other by Tukey test (*P* ≤ 0.05).

Fig 3 depicts the comparative analyses of the RF at the end of each phase of the thermal challenge test: before thermal challenge, the actual challenge, and after the challenge for each age and treatment. Independent of the ages and treatments, almost all broilers increased their RF when thermally challenged and then reduced their frequencies when returned to their preferred rearing temperatures after thermal challenges to values similar to those observed before thermal challenge. This observation was not seen for broilers from eggs incubated at 39°C with Short-Long HP at the 2^nd^ day of age (Fig 3D).

### Variation of the rectal temperature in the three phases of the thermal challenge test

**Fig 4:**
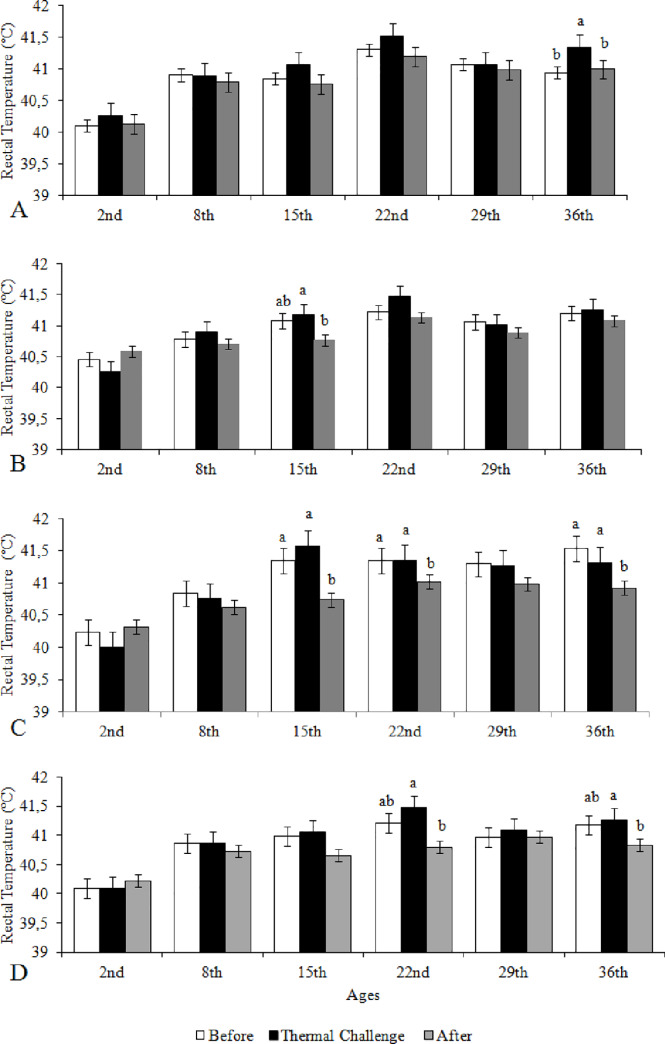
Rectal temperature of broilers from eggs incubated at 37.5°C (A,B) and 39°C (C, D) with Long-Short (A, C) and Short-Long (B, D) hatching pattern, obtained at the preferred temperature before thermal challenge, at thermal challenge (5°C above thermal preference for 45 min), and upon return to thermal preference after thermal challenge, according to age. Short-Long and Long-Short: time intervals between pippings and between external pipping and hatching (2–8,2+20-26h and 11–18+6-10h, respectively). a-b: means with similar letters do not differ from each other by Tukey test (*P* ≤ 0.05).

In Fig 4 we compare the rectal temperatures of broilers in the first phase of the thermal challenge test, at the end of the actual thermal challenge, and in the final phase of the challenge within each age and for each treatment. Broilers from eggs incubated at 37.5°C with Long-Short HP did not change their rectal temperatures during the thermal challenge test up to the 29^th^ day of age. However, at day 36, their rectal temperatures increased with the thermal challenge and then returned to the temperatures they exhibited before the challenge when the broilers were exposed once again to their preferred rearing temperatures (Fig 4A). For broilers with Short-Long HP whose eggs were incubated at 37.5°C, we found that significant variations in rectal temperature during the thermal challenge test occurred only at the 15^th^ day of age, when they reduced their rectal temperatures once they were returned to their preferred rearing temperatures after the challenge (Fig 4B). The thermal challenge test did not affect the rectal temperatures of broilers with both Long-Short and Short-Long HP derived from eggs incubated at 39°C. However, when the birds were exposed back to their preferred rearing temperature after thermal challenge, we observed reduction of their rectal temperatures at days 15, 22, and 36 for the Long-Short and at the days 22 and 36 for the Short-Long HP broilers (*P* ≤ 0.05) (Fig 4C and 4D).

### Effects of HP and IT on broiler performance

The results relative to broiler performance are shown in the Table 5. Broilers with Short-Long and Long-Short HP presented similar feed intake, but the former had better feed conversion that resulted in higher weight gain. Broiler from egg incubation at 39°C compared to broilers from eggs incubated at 37.5°C had lower feed comsumption, resulting in worse feed conversion, weight gain, and body weight. No birds died during the rearing period.

**Table 5:** Overall performance of broilers with Short-Long and Long-Short hatching pattern obtained from eggs submitted to usual (37.5°C) or high (39°C) incubation temperature from day 13 of incubation till hatching.

Treatments	Feed intake^2^	Weight gain^2^		Feed conversion^2^ (g/g)	Body weight^3^ (g)
	(g)	(g)			
Hatching Pattern (HP)^1^					
Long-Short	5,404±225	2,698±303^b^		2.01±0.16^a^	2,721±326^b^
Short-Long	5,436±168	2,801±258^a^		1.95±0.13^b^	2,824±280^a^
Incubation Temperature (IT)					
37.5°C	5,549±165^a^	3,008±118^a^		1.84±0.63^b^	3,054±118^a^
39°C	5,296±140^b^	2,506±105^b^		2.11±0.57^a^	2,506±104^b^
*P*-value					
HP	0.5919	0.0113		0.0020	0.0113
IT	0.0002	<0.0001		<0.0001	<0.0001
HP x IT	0.2450	0.1428		0.1543	0.1431
CV (%)	2.84	3.55		2.58	3.53

CV: coefficent of variation.

^1^Time intervals between pippings and between external pipping and actual hatching (Short-Long: 2-8h10min+20-26h, Long-Short: 11–18+6-10h).

^2^Performance from day 1 to 42 of age.

^3^Weight at the 42^nd^ day of age.

^a-b^: means followed by the same superscript letter in the columns do not differ from each other (*P* ≤ 0.05).

## Discussion

This study ahowed for the first time that thermal preference, heat tolerance and performance during rearing of Cobb 500 male broilers are affected by HP with or without interaction with IT, confirming our previous hypothesis.

When eggs were incubated at 37.5°C, broilers with Short-Long HP preferred higher ambient temperature than those with Long-Short HP during whole rearing period. However, when egg incubation occurred at 39°C, broilers with Short-Long HP preferred higher rearing temperatures after the 21^st^ day of age. Our data suggest that broilers with Long-Short and Short-Long HP should be reared under distinct ambient temperatures. In a previous study, we observed that broiler hatchlings derived from eggs incubated at 37.5°C and with Long-Short HP presented lower RBC (counts of blood red cells) and HGB (hemoglobin concentration) than those with Short-Long HP [25]. We also found broiler hatchlings from eggs incubated at 39°C had higher HCT (hematocrit) and MCH (mean corpuscular hemoglobin) values than hatchlings from egg incubated at 37.5°C [25]. Considering that these erythrocyte variables determine the bird potential for blood gas exchange [34, 35, 36], it is plausible that the preference for lower or higher ambient rearing temperatures results from lesser or greater blood potential for gas exchange, respectively. However, whether there is a relationship between thermal preference and erythrocytic values during the rearing period remains to be investigated. Further, broiler hatchlings of eggs incubated at 39°C presented rectal temperature 1.21°C lower than hatchlings of eggs incubated at 37.5°C. According to previous study [24], broiler hatchlings of eggs incubated at 39°C have thinner and more vascularized skin than hatchling of eggs incubated at 37.5°C, which may indicate that the former have a higher heat loss potential by conduction than the latter. However, egg incubation at 39°C had no effect on the absolute and relative feathering weight, as well as on barb number, length, and width, and barbule length of the hatchlings [24]. Thus, it is possible that the preference for higher ambient rearing temperatures and the lower rectal temperatures found in hatchlings from egg incubated at 39°C (our data) are due to greater heat loss by conduction through the skin. Our results agree with those in turkey, which showed that these birds prefer higher ambient rearing temperatures when their eggs were exposed to 38.5°C after the 7^th^ day of incubation [21]. In contrast, chicks of the layer strain (Lohmann) submitted to intermittent heat stress (4h/day at 40°C) from the 14^th^ to the 18^th^ day of incubation prefered lower ambient rearing temperature up to the 7^th^ day of life [22]. These differences may be related to the specific metabolism of each strain as well as the difference between the intensity and duration of the IT changes. It is noteworthy that independently of the HP and IT, broilers preferred lower rearing temperatures than those recommended for the chick strain [28].

The current broiler chicken lines selected by breeders for greater weight gain and faster growth present high metabolic heat production, which imposes a need heat loss to maintain body temperature. Heat transference only occurs from a warmer to a colder ambient temperature [37]. Thus, because of the current strains increased metabolic heat production, the ambient temperature should be lowered throuthout the rearing period for heat loss to occur. It is known that when rearing temperature elevates, broilers reduce their feed consumption and activity to decrease their metabolic heat production. At the same time, they increase the RF, as well as peripheral blood circulation and water intake to further promote their heat loss [38, 39]. However, if the rearing temperature reaches values above of those tolerated by broilers, high or total mortality occurs. Thermal challenge has been used in previous study to test thermotolerance induced by incubation temperature manipulation in poultry [23, 40, 41, 42]. Egg exposure to 39.5°C for 3h during distinct days of embryogenesis (E8- E10 and E16 to E18) did not prevent hyperthermia (average Tb>44.3°C) during the thermal challenge (5°C for 6h on day 42 od age) [40]. Egg incubation at 39.5°C and 65% RH from embryonic day 7 to 16, either continuously (24 h) or intermittently (12 h), did not prevent hyperthermia, but promoted a significant improvement in the acquisition of thermotolerance in broilers subjected to thermal challenged (35°C for 5 h, at day 35 of age), which was evidenced by the lower level of plasma corticosterone [41]. Higher heat tolerance induced by incubation temperature manipulation (12 h/d, 39.5°C, 65% RH, from day 7 to 16 of incubation) was also evidenced by absence of an increase in the heterophil/lymphocyte ratio during heat challenge (5 h, 32°C, at day 34) [42]. Also, egg incubation at 39°C from day 13 did not prevent hyperthermia during heat challenge (45min, 5°C above thermal preference) at day 21–22 and 28–29 of age [23]. Thermal challenge was used in this study to verify whether broilers with Long-Short and Short-Long HP respond diferently to heat exposure during rearing and whether incubation at 39°C during fetal phase made these birds more tolerant to heat. Our results show that, regardless of the IT and HP, broilers subjected to thermal challenge (5°C higher than the preferred rearing temperature) presented increased RF and restored it to the pre-challenge frequencies in all analyzed ages without changing their rectal temperature in all analyzed ages, except at the 2^nd^ and 36^th^ day of age. At the 36^th^ day of age, broilers of eggs incubated at 37.5°C with Long-Short HP increased rectal temperature in response to thermal challenge. In contrast, at the 2^nd^ day of age, broilers of eggs incubated at 39°C with Short-Long HP did not alter their RF in response to thermal challenge and, consequently, maintained their rectal temperature. These findings indicate that all broilers, independently of the IT and HP, used heat loss by evaporation to maintain their body temperature, but that the 36 day-old broilers derived from eggs incubated at 37.5°C with Long-Short HP failed to maintain body temperature. Adittionally, the absence of a thermal challenge effect on hatchlings of eggs incubated at 39°C with Short-Long HP may be related to these chicks’ higher body temperatures and suggest that the sudden increase of ambient temperature (5°C higher than the preference) did not consist a real challenge for these hatchlings. Our results show high incubation temperature from day 13 of incubation did not change thermotolerance in chicks that have Short-Long and Long-Short hatching pattern.

Although feed consumption had not been affected by HP, broilers with Short-Long HP showed better feed conversion and, consequently, greater weight gain during the rearing period and body weight at 42 days of age compared to broiler with Long-Short HP. Further, broilers of eggs incubated at 39°C reduced feed consumption and worsened feed conversion resulting in lower body gain and body weight. The worse performance of broilers with Long-Short HP and of broilers derived from eggs incubated at 39°C suggests that the rearing temperatures used in the present study [28] were not those that would foster the best performance, because the birds preferred different rearing temperatures. Our results are in agreement with previous works, in which high incubation temperature (39°C from day 13 or 39.5°C and 65% RH for 12 h/d from E7 to E16 inclusive) had negative effects on the broiler performance [43, 44].

In summary, the data presented herein show that HP associated or not with high IT during fetal phase affect the broilers’ thermal preference and performance throuthout the rearing period. Broilers with Long-Short HP prefer lower temperatures than broilers with Short-Long HP during whole rearing period when the eggs were incubated at 37.5°C and from the 21^st^ day of age when the eggs were incubated at 39°C. Incubation at 39°C from fetal phase increased the preferred rearing temperature of broilers with Long-Short and Short-Long HP_,_ but this effect was observed in the latter after the 21^st^ day of age. Further, better performance was observed for broilers with Short-Long HP and for broilers of eggs incubated at 37.5°C than for broilers with Long-Short HP and for broilers of eggs incubated at 39°C with Short-Long HP.

Our findings show that Cobb500 broilers with Long-Short and Short-Long HP should be raised apart. Today, however, separating Long-Short and Short-Long hatching pattern chicks within a hatcher is not practical. The results also show that the rearing temperature suggested for the lines should be revised. Indeed, taking into consideration the duration of the hatching period, broilers of the line used herein (Cobb 500) preferred a lower rearing temperature than the prescribed by the Management Guide for Broiler [28].

The demand for animal protein is expected to rise by 70–80% in 2050 and therefore we face an enormous challenge to feed the 9 billion people the earth is expected to hold by then [45]. Poultry-derived protein will certainly play a major role in meeting this demand but every effort will count to raise broiler performance and production results. Therefore, the data presented here may be of great interest to companies (hatcheries) to develop new incubation management strategies and obtain more homogeneous chick batches with improved performance and production.

## Conclusion

Altogether our results are consistent with the hypothesis that hatching phase is an important factor influencing chick thermal preference and that high incubation temperature from day 13 affects the hatching pattern effects. Our findings demonstrate, for the first time, that the hatching pattern associated or not to high incubation temperature influence the Cobb 500 male broiler thermal preference and heat response throughout the rearing period. From a production point of view, the results indicate that chicks with Long-Short and Short-Long HP should be reared separately. However, in practice, the handling becomes difficult when using open sheds and even closed sheds that do not have efficient control of room temperature.
